# Supplementary material for: Jujube Powder Enhances Cyclophosphamide Efficiency against Murine Colon Cancer by Enriching CD8+ T Cells While Inhibiting Eosinophilia
Source: Nutrients. 2021 Aug 4;13(8):2700. doi: 10.3390/nu13082700 (PMC8401958; doi:10.3390/nu13082700)
Supplement: Supplementary file 1 [file nutrients-13-02700-s001.zip › nutrients-1316780-supplementary.pdf]

Supplementary Material

Table S1 Primers used for real-time PCR

Primer		Sequence
IL7	Forward	5′-CTGATGATCAGCATCGATGAATTGG-3′
	Reverse	5′-GCAGCACGATTTAGAAAAGCAGCTT
IL-3	Forward	5′-TTTCGGAGAGTAAACCTGTCCA-3′
	Reverse	5′-AGGCAGGCAACAGTTAAGTTTC-3′
GM-CSF	Forward	5′-AAAAACAGCCAGTGTCCTGTG-3′
	Reverse	5′-GATGCTGACGTTCTTGGGAAG-3′
M-CSF	Forward	5′-TCCAACCTTACTTCAAAGGGGA-3′
	Reverse	5′-AGGCTCGAAAGGAGTTATACCA-3′
TPO	Forward	5′-CTTGGAGCTATGGCAATAATGCT-3′
	Reverse	5′-GAGCTGGCTCGTTTCCACA-3′

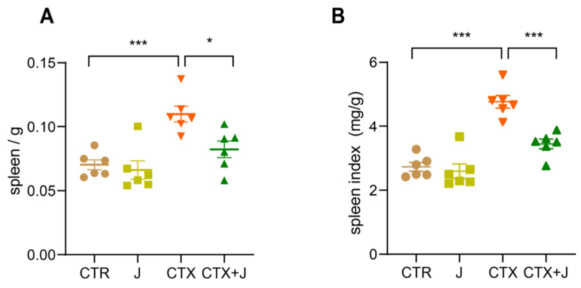

Figure S1 (**A**) spleen weight (**B**) spleen index of different group

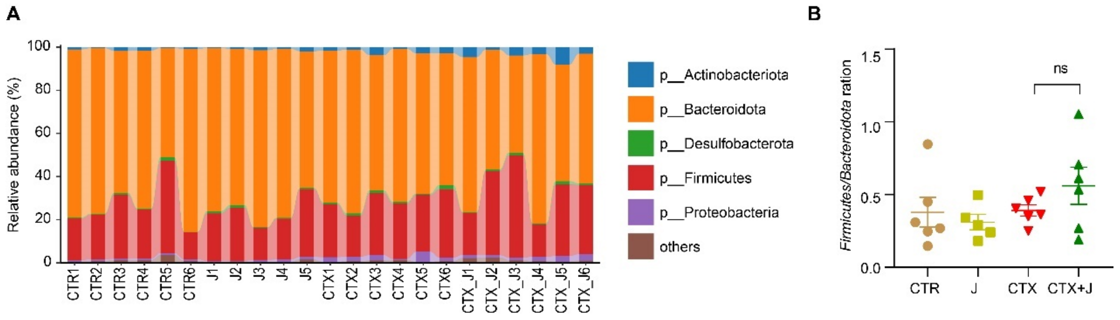

Figure S2 (**A**) Phylum level composition of representative bacterial in each group (**B**) The *Firmicutes* to *Bacteroidota* ratio

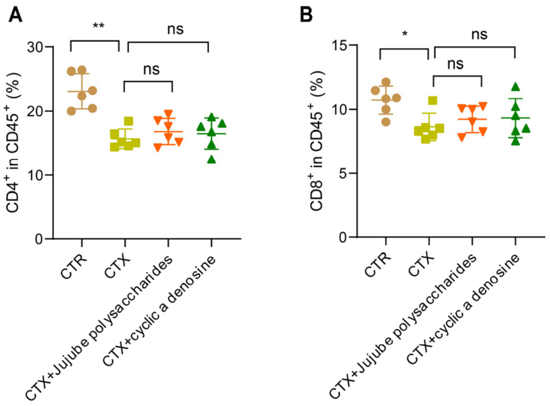

Figure S3 The effect of jujube polysaccharides and cyclic adenosine on CTX treatment (**A**) The ratio CD4^{+} T cells (**B**) CD8^{+} T cells of CD45^{+} cells in peripheral blood.
